# Focus group interview as a research method in the development of the e-learning in suicide prevention for students in poland

**DOI:** 10.1192/j.eurpsy.2021.188

**Published:** 2021-08-13

**Authors:** R. Pawelec, A. Kwiatkowska

**Affiliations:** Media Dept., Uniwersytet Warszawski, Warszawa, Poland

**Keywords:** Suicide, focus group interview, student, media

## Abstract

Project ELLIPSE (E-LifeLong Learning in Prevention of Suicide in Europe) was developed in response to need for preventive suicide programs. It was noticed that it is important for academic teachers and students, especially those who teach and study in the fields of medicine, nursing, psychology, sociology or those related to the media. The goal is creating an e-learning platform for students and teachers on suicide prevention along with a textbook and a free self-help application. For this purpose the milestone was the conducting of the focus group interviews in Poland. Participants were recruited from the above mentioned groups (students and professionals). The objectives of this part of the project were among others: raising awareness of the problem of suicide and prevention, giving good examples on suicide prevention in working life, preparation of a program proposal for e-learning content. Students interest in the problems of suicidology was high and they comes optimistic to the idea of prevention, they want to know especially about how to help somebody, how to talk and not to harm people and something more in their future profession. Only theoretical approach will be not enough for them, the rules of how to NOT react, will not be interesting because they know something about it. They showed a knowledge of basic concepts, extensive and multi-context knowledge of causes and warning signals and great understanding for suicides of all ages. However, they themselves were uncertain how to help such a person, and showed poor recognition of institutional help.

**Disclosure:**

No significant relationships.

